# Brain and Retinal Abnormalities in the 5xFAD Mouse Model of Alzheimer's Disease at Early Stages

**DOI:** 10.3389/fnins.2021.681831

**Published:** 2021-07-23

**Authors:** Mengrong Zhang, Liting Zhong, Xiu Han, Guoyin Xiong, Di Xu, Sensen Zhang, Haiyang Cheng, Kin Chiu, Ying Xu

**Affiliations:** ^1^Guangdong-Hongkong-Macau Institute of Central Nervous System Regeneration, Jinan University, Guangzhou, China; ^2^Department of Ophthalmology, LKF Faculty of Medicine, The University of Hong Kong, Hong Kong SAR, China; ^3^State Key Laboratory of Brain and Cognitive Sciences, The University of Hong Kong, Hong Kong SAR, China; ^4^Key Laboratory of Central Nervous System Regeneration, Jinan University, Ministry of Education, Guangzhou, China; ^5^Co-Innovation Center of Neuroregeneration, Nantong University, Jiangsu, China

**Keywords:** Alzheimer's disease, retinal ganglion cell, multielectrode array, long-term potentiation, photoreceptor

## Abstract

One of the major challenges in treating Alzheimer's disease (AD) is its early diagnosis. Increasing data from clinical and animal research indicate that the retina may facilitate an early diagnosis of AD. However, a previous study on the 5xFAD (a fast AD model), showing retinal changes before those in the brain, has been questioned because of the involvement of the retinal degeneration allele Pde6b^rd1^. Here, we tested in parallel, at 4 and 6 months of age, both the retinal and the brain structure and function in a 5xFAD mouse line that carries no mutation of rd1. In the three tested regions of the 5xFAD brain (hippocampus, visual cortex, and olfactory bulb), the Aβ plaques were more numerous than in wild-type (WT) littermates already at 4 months, but deterioration in the cognitive behavioral test and long-term potentiation (LTP) lagged behind, showing significant deterioration only at 6 months. Similarly in the retina, structural changes preceded functional decay. At 4 months, the retina was generally normal except for a thicker outer nuclear layer in the middle region than WT. At 6 months, the visual behavior (as seen by an optomotor test) was clearly impaired. While the full-field and pattern electroretinogram (ERG) responses were relatively normal, the light responses of the retinal ganglion cells (measured with multielectrode-array recording) were decreased. Structurally, the retina became abnormally thick with few more Aβ plaques and activated glia cells. In conclusion, the timeline of the degenerative processes in the retina and the brain is similar, supporting the use of non-invasive methods to test the retinal structure and function to reflect changes in the brain for early AD diagnosis.

## Introduction

Alzheimer's disease (AD) is a progressive, age-related neurodegenerative disorder that causes memory loss and a decline in cognitive function in patients. It is characterized by abnormal accumulation of beta-amyloid (Aβ) plaques and neurofibrillary tangles in the central nervous system, which cause selective loss in neurons and synaptic connections (Huang and Jiang, 2009). As the current standard tests including testing Aβ biomarkers by positron emission tomography and cerebrospinal fluid assays are invasive and expensive (Sutphen et al., 2014), early diagnosis of AD remains a challenge for its treatment.

Increasing data from clinical and animal research indicate that retina may serve as a window for early diagnostic of AD. Several changes were reported in AD patients' vision; these include decrease in visual acuity, contrast sensitivity, color discrimination, pattern electroretinogram (pERG) response, and defects in the visual fields. Morphological changes were also shown with optical coherence tomography (OCT) scanning, and these include a thinning of the retinal nerve fiber layer (RNFL) and a deficit in the retinal vasculature [reviewed in Chiquita et al. (2019)]. Visual and morphological deficits including a decrease in pERG response, presence of Aβ and Tau tangles in retina, thinning of RNFL and ganglion cell layers, loss of ganglion cell numbers, and reactive gliosis [reviewed in Chiquita et al. (2019)] were reported in different transgenic AD animal models at various degrees. Furthermore, simultaneous examinations of the retina and brain pathologies are limited, and hence, the correlation of the time course of retinal pathology and brain degeneration remained unclear.

The 5xFAD mice is a good model with emphasis on the overaccumulation of Aβ with marked AD symptoms seen in behavioral tests by the age of 6 months (Oakley et al., 2006). Using this model, it has been reported that the Aβ deposits in the retina appears as early as 1.5M (Pogue et al., 2015), and retinal light responses and visual acuity decays earlier than the cognitive deficit (Criscuolo et al., 2018). However, the 5xFAD mice used in these studies contained Pde6b^rd1^ mutation that cause rods to die; hence, the effect of the 5xFAD mutations could not be isolated. Therefore, it is important to reassess the progressive retinal degeneration that is caused by Aβ overaccumulation. Work done by Lim et al. (2020) found abnormal retinal structure and function at age of 6, 12, and 17 months. These retinal degenerative processes were progressive and associated with amyloid pathology patterns similar to that of the brain. However, it remained unclear whether the retina shows abnormalities at an early stage of AD or even precedes the brain degeneration in this non-Pde6b^rd1^ 5xFAD mice. Therefore, the goal of this study was to investigate the progressive changes in the retinal structure and function of 5xFAD mice with no mutation of rd1 and to compare them with Aβ-related pathologies in the brain.

## Materials and Methods

### Animals

Transgenic mice with 5xFAD mutations [B6.Cg-Tg (APPSwFlLon, PSEN1^*^M146L^*^L286V) 6799Vas/Mmjax] (Oakley et al., 2006) were purchased from Jackson Lab (MMRRC stock no. 34848) with no retinal degeneration allele Pde6b^rd1^. 5xFAD transgenic mice overexpress both mutant human APP (695) with the Swedish (K670N, M671L), Florida (I716V), and London (V717I) Familial Alzheimer's Disease (FAD) mutations and human PS1 harboring two FAD mutations, M146L and L286V, under transcriptional control of the neural-specific mouse Thy1 promoter (Oakley et al., 2006). C57BL/6J female mice were purchased from Guangdong Medical Lab Animal Center to be bred to 5xFAD to maintain the colony. Hemizygous 5xFAD mice and non-transgenic wild-type littermates were used. All animals were kept under standard laboratory conditions with 12-h/12-h light/dark cycles and were supplied with regular food and water. All animal procedures were performed according to the ARRIVE guidelines and were approved by competent ethics committee at Jinan University. All efforts were taken to minimize the number of animals used and their suffering.

### Morris Water Maze Test

A water tank 70 cm in diameter and 35 cm in height was filled with water to 16.5 cm at 22–25°C. The pool was divided into four equal quadrants. A 4×4-cm^2^ white escape platform was placed 5 cm beneath the water at the center of the fourth quadrant. During four consecutive days of training session, mice were placed into the pool and allowed to search for the platform for 60 s for four trials (once from each quadrant) with at least 10-min interval. Animals were guided to the platform if they could not find it within 60 s, in which case the latency was recorded as 60 s. On the fifth day, the platform was removed from the pool, and mice were allowed to swim freely in the pool for 60 s. Times of animals traversing the original platform position and the time spent in the target quarter were measured to evaluate the working memory of the animal. Data were recorded with a video camera and analyzed using EthoVision XT 7.0 (Noldus, Wageningen, the Netherlands). Animals that refused to swim were excluded from the experiments.

### Visual Behavioral Tests

The day after the water maze test, the visual performance of these mice was tested by a black–white transition system and then an optokinectic system. The black–white transition system measures the ability of animal to tell luminance. As we previously described (Zhang et al., 2017), an animal was placed at the center of the white chamber that was connected with the black chamber and was able to move freely between these two chambers. The time at which the animal stayed in the black chamber was recorded by Noldus EthoVision XT 8.0 software.

Optomotor system measured the visual acuity of the animal. Briefly, dark-adapted mice were placed on a pedestal located at the center of an enclosure formed by four video monitors that displayed the stimulus gratings. Vertical sine wave gratings (100% contrast) written in MATLAB (MathWorks, Natick, MA, USA) were projected on the computer monitors and rotated at the speed of 12°/s with increasing spatial frequencies of 0.1, 0.2, 0.3, 0.35, 0.4, 0.45, 0.5, and 0.6 cycle/degree. For each spatial frequency, the grating was rotated clockwise for 1 min and then counterclockwise for another 1 min. Animals reflexively track the gratings by head movements as long as they could follow them. The head movements were videotaped, and the maximal spatial frequency at which an optokinetic response could be followed was recorded as the visual acuity of the animal.

### Electroretinogram

After behavioral tests, mice were dark adapted for 4 h, and the electroretinogram of mice was measured to test retinal function with a RETI-scan system (Roland Consult, RETI-scan, Heidelberger, Germany) as we previously described (Zhang et al., 2017; Liu et al., 2018). Briefly, mice were anesthetized with tribromoethanol (0.14 ml/10 g bodyweight of 1.25% solution) and placed on a heated platform (37°C) under dim red light. Pupils were dilated with phenylephrine HCl (0.5%) and tropicamide (0.5%). ERGs were recorded with gold-plated wire loop electrodes contacting the corneal surface as the active electrode. Stainless steel needle electrodes were inserted in the skin near the eye and in the tail serving as reference and ground leads, respectively. Dark-adapted animals were stimulated with full-field green flashes of graded intensities of 0.01, 0.1, and 3.0 cd/m^2^ by Ganzfied stimulator. Then, mice were light adapted for 5 min under bright green background (20 cd/m^2^), and photopic responses to green flashes of 3.0 and 10.0 cd/m^2^ were recorded. ERG data were collected with RETI-scan system at a sampling rate of 2 kHz and analyzed with the RETIport software (Roland) after 50 Hz low-pass filtering. To isolate the oscillatory response (OPs), a 100-Hz high-pass filter was further applied. The a-wave amplitude was measured from baseline to the first negative peak, and the b-wave amplitude was measured from a-wave trough to the next positive peak. Photopic negative response (PhNR) was measured as the amplitude of the negative peak following b-wave relative to the baseline. To measure the amplitude of OPs, the voltage difference between the second negative peak (N2) to positive peak (P2) and between the third negative peak (N3) to positive peak (P3) were measured. For each animal, the average response of the two eyes was taken as one data point. To further evaluate the function of retinal ganglion cells, some mice were tested with patterned ERG projected by a flat LED screen (Roland). The pattern consisted of a horizontal grating (with 0.5°/cycle and 99% contrast) and a flicker checkerboard (horizontal grating size, 2°50″; checkerboard size, 4°15″; flickering frequency, 1.0 Hz). The animal was tilted with the left eye covered and the right eye directly facing the center of the screen at the distance of 26 cm for the recording after photopic ERG recording. The sampling rate was 1 kHz, and 200 trails were recorded and averaged. The pERG waveform is characterized by a small initial negative wave N1, followed by a large positive wave P1, and then a second negative wave N2. The amplitude of P1-N2 peak was measured to evaluate pERG.

### Multielectrode Array Recording From Retina and Data Collection

To examine the light response of single ganglion cell, multielectrode array (MEA) recording was performed on whole-mount retinas as we previously described (Liu et al., 2018; Bao et al., 2019). Briefly, mice were dark adapted for 3 h before euthanization, and a small piece of retina (~2×2 mm^2^) from whole-mount regions avoiding main blood vessels was pressed down on an 8×8 MEA array (with electrodes of 20 μm in diameter spaced 100 μm apart; P210A, Japan) by a platinum ring to obtain a close contact between the ganglion cells and the electrodes. The MEA array with the retina was transferred to the recording stage, connected to the amplifier (MED64 amplifier; Alpha MED Scientific, Inc., Osaka, Japan), and perfused continuously with the oxygenated AMES solution at a rate of 3 ml/min at ~32°C. After dark adaptation in a light-tight enclosure for over 30 min, retinas were stimulated with a white light-emitting diode (LEDWE-15; Thorlabs, Newton, NJ, USA) with the stimulation intensity and duration controlled by the main amplifier (MED64; Alpha MED Scientific, Inc.). The LED gave a full-field flash focused onto the photoreceptor layer of the retina. The flash protocol consisted of a 2-s light ON with a saturating intensity of 3.6 × 10^7^ photons/μm^2^/s, followed by an 8-s light OFF, and repeated 30 times.

The MEA system with MED64 amplifier (Japan) and Mobius software (MED64, Japan) was used for recording and filtering spike trains from each of the electrode in the array. Extracellular spikes were bandpass filtered between 100 and 5,000 Hz, digitized at a rate of 20 kHz, and subsequently analyzed offline.

To identify responses from each individual cell, the MEA data were processed offline using a spike sorter software (Offline Sorter, Plexon Inc., Dallas, TX, USA) as previously described (Liu et al., 2018). Sorted spikes were then exported to Spike2 (version 8, CED, UK), MATLAB (MathWorks), and R software (version 3.3.0) to get the peristimulus time histograms (PSTHs) and raster plots of individual cells with a 10-ms bin width. Light responses were measured as the average spike rate during the first 2 s of light onset (ON) or offset (OFF) or the average of both ON and OFF (for ON–OFF), subtracted by the spontaneous spiking (average response within 2 s before light onset).

### LTP Recordings With Microelectrode Array

LTP was recorded from CA3–CA1 regions of hippocampal slices from 6-month-old 5xFAD and WT mice. Briefly, a mouse was decapitated under isoflurane anesthesia; then, the brain was quickly dissected and placed in ice-cold oxygenated (95% O_2_/5% CO_2_) sucrose cutting solution (containing in mM: 40 NaCl, 4 KCl, 26 NaHCO_3_, 1.25 NaH_2_PO_4_, 0.5 CaCl_2_,7 MgCl_2_, 10 D-glucose, and 150 sucrose, pH 7.4, 330 mOsmol). Slices were then cut into 300 μm thickness with a Vibratome (VT1000S; Leica, Wetzlar, Germany) and maintained at room temperature for at least 1 h in the oxygenated artificial cerebrospinal fluid (ACSF) (comprising in mM: 125 NaCl, 3.5 KCl, 26 NaHCO_3_, 1.2 NaH_2_PO_4_, 2.4 CaCl_2_, 1.3 MgCl_2_, 25 D-glucose, pH 7.35, 310 mOsmol). Then, a single slice was transferred to an 8×8 MEA array (with electrodes of 50 μm in diameter spaced 200 μm apart; Japan), pressed down by a nylon mesh, and continually perfused with oxygenated ACSF buffer (flowrate, 3 ml/min) at 34°C. One of the microelectrodes under the apical dendritic region of CA3 was selected for stimulating the Schaffer collateral pathway. A biphasic electric current (ranging from −10 to 40 pA) of 0.20 ms was given every 20 s at the stimulus intensity sufficient to elicit 30–50% maximal extracellular field excitatory postsynaptic potential (fEPSP) recorded from other electrodes. After establishing a stable baseline for at least 15 min, three repeated theta-burst stimulations (TBSs) were applied. Each TBS contained 10 trains of four 100-Hz pulses at 5 Hz, and TBS was repeated three times with a 20-s interval. After the TBS stimuli, fEPSP were recorded every 20 s for another 45 min. The peak amplitudes of the fEPSPs were measured by the MED64 Mobius software.

For both retinal and brain slice recording, at the end of recording, the position of the tissue on the array was verified under a dissecting microscope, and a bright field image was taken with a digital camera (Mshot Image Analysis System; MC16, Guangzhou, China).

### Tissue Processing and Immunocytochemistry

Animals were killed by anesthetic overdose with tribromoethanol, and eyes were enucleated and fixed in 4% paraformaldehyde (PFA) for 30 min at 4°C. Following fixation, the eyes were rinsed in 0.01 M phosphate buffered saline (PBS), cryoprotected overnight at 4°C in 0.01 M PBS containing 30% sucrose, and embedded in optimal cutting temperature compound (OCT; Tissue Tek, Torrance, CA, USA). Retinas were cryosectioned through the optic disk (OD) longitudinally at a thickness of 15 μm, and sections were mounted on glass slides for future process.

For brain tissue collection, brains were removed and washed in PBS three times with 1 min each time, then fixed in 4% paraformaldehyde (PFA) for 24 h at room temperature (RT). Then, brains were rinsed in PBS, cryoprotected in 0.01 M PBS containing 10, 20, 30, and 40% sucrose at 4°C overnight for each concentration before embedded in OCT. Brain were cryosectioned into sagittal slices at a thickness of 20 μm, and sections were mounted on glass slides for future process.

For immunochemical staining, both eyes and brain sections were washed three times for 5 min with 0.1% Triton X-100 in PBS (0.1% PBST) and incubated in 0.3% PBST containing 3% normal donkey serum (NDS), 1% bovine serum albumin (BSA), and 0.3% Triton X-100 for 1 h at RT, then incubated with primary antibodies overnight at 4°C. After thorough washes with 0.1% PBST, retinal or brain sections were incubated with secondary antibodies for 1 h at RT. Sections were then washed, mounted, and sealed under coverslips. For 4′,6-diamidino-2-phenylindole (DAPI) staining, sections were incubated with DAPI (1:1,000, Electron Microscopy Sciences, Hatfield, PA, USA) for 5 min at room temperature, then washed before mounting.

The primary antibodies used were rat anti-glial fibrillary acidic protein (anti-GFAP) (1:500, 13-0300, Thermo Fisher, Waltham, USA), rabbit anti-Iba1 (1:1,000, 019-19741, Wako, Osaka, Japan); anti-Brn3A (1:500, ab81213, Abcam, Cambridge, UK) and Aβ 1-42 antibody (1:1,000, Cat. # AB5078P, Millipore, Burlington, USA). Secondary antibodies used were donkey-antirabbit or goat-antirat IgG (conjugated to Alexa 488 or 594; 1:1,000, Invitrogen, Waltham, USA).

To calculate the thickness of each retinal layer, retinal slices were stained with Hematoxylin and Eosin (HE) Staining Kit (G1120, Solarbio, Beijing, China) according to the provided protocol. To stain for senile plaques, brain sections were rehydrated and dehydrated in distilled water for 2 min. Then, they were incubated with thioflavin solution (1% in DDW) for 5 min. Slices were immersed in 70% alcohol for 5 min and washed with distilled water two times before mounted.

### Image Collection and Processing

Fluorescent or immunostaining images were captured using a Zeiss LSM700 confocal microscope or fluorescent microscope (Carl Zeiss, Oberkochen, Germany). To measure the survival of retinal ganglion cells, Brn3a-positive cells were counted on whole-mount retina from 12 regions (field size, 200×200 μm) distributed at a distance of 300, 1,000, and 1,700 μm from the optic disk for each quadrant of the retina, and the average density of RGCs over 12 regions was calculated. To measure the Iba1 expression, the number of Iba-1-positive cells were counted from the middle region on the whole-mount retina at the size of 320×320 μm. For all retinal slices, to ensure analysis of the same eccentricity for different retinas, images were taken from regions 1–1.2 mm from the optic disk. To measure the thickness of each layer of retina, a line was drawn from the left, right, and center of each HE staining image, and the length of three lines were measured and averaged as one data point for the image. To measure the fluorescent intensity of GFAP, retinal sections from different groups were processed simultaneously with the same procedure and imaging parameters; the mean fluorescent intensity in the inner retinal region (INL, IPL, and GCL) was measured by Zen software (Zeiss, Germany), then normalized to the mean of WT at the same age. To quantify the number of Aβ in brain regions, the hippocampal area (HP, 1,500×2,250 μm), visual cortex (VC, 1,100×850 μm), and ventro-posterior of olfactory bulb (OB, 1,100×850 μm) were imaged and analyzed from the sagittal plane.

Image J software (NIH, Bethesda, MD, USA) was applied for analyzing all the measurements. For each retina and brain slice, data from three to five images was averaged to provide one data point; these were then averaged for all retinas to provide the average of each group. For a better display of the images in the figures, intensity enhancement was applied by Photoshop (Adobe Inc., San Jose, USA) with the same adjustment.

### Statistical Analysis

All data are expressed as mean ± SEM. Student's *t*-test or two-way ANOVA with Sidak's *post-hoc* tests was performed with GraphPad 7 (GraphPad Software, San Diego, CA, USA) depending on the number of groups to compare. *p* < 0.05 were considered statistically significant, and *p* < 0.01 were highly significant. Unless otherwise stated, the “n” indicates the total number of mice examined for each group.

## Results

### 5xFAD Mice Demonstrate a Deficit in Working Memory and Hippocampal LTP at 6 Months

**Figure 1:**
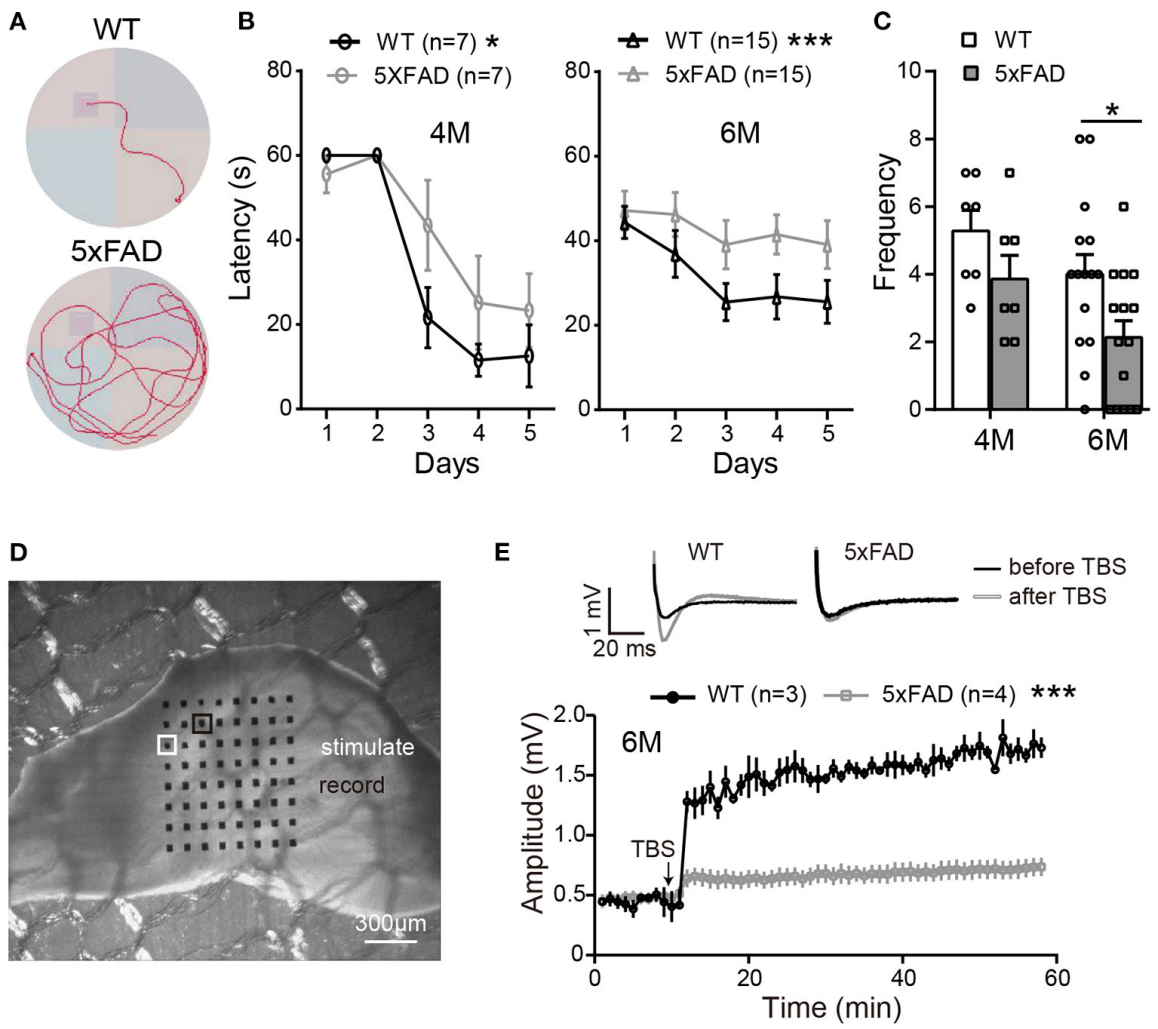
The working memory and LTP deficiency in 5xFAD mice. **(A)** Swimming traces of a WT and 5xFAD mice in the water maze, with the hidden platform in the upper-left quadrant. **(B)** The latency for mice to reach the platform during the first 5 days of trials at 4 and 6 months. **(C)** The frequency of mice crossing the platform region on the test day. 5xFAD mice at 6 months took longer time to locate the platform during the trial day and crossed the platform region less frequently than WT. **(D)** Illustration of a mouse hippocampus slice placed on the 8×8 multielectrode array. One of the electrodes (white squared) was used to apply the electric stimuli; the other electrodes recorded the evoked field potential; the electrode squared in black, which gave the best response, was selected for data analysis. **(E)** Average changes in the amplitude of the evoked post-synaptic potential (EPSP) within 45 min after the theta-burst stimulation (TBS) in WT and 5xFAD mice. Typical EPSP of a WT and 5xFAD mice before (black line) and after (gray line) TBS stimulation at an expanded time scale are shown above. n, number of experimental animals. Data shown as mean ± SEM. *p < 0.05; ***p < 0.001; two-way ANOVA.

In order to identify the behavioral changes in our 5xFAD mice, we first performed a Morris water maze test for cognitive performance. Mice were tested at the ages of 4 and 6 months (examples of the moving traces at 6 months are shown in Figure 1A). During trial days, both WT mice and 5xFAD showed a decrease in the escape latency, but the latency of 5xFAD mice decreased more slowly than WT (*p* < 0.05 for 4 months and *p* < 0.001 for 6 months, two-way ANOVA) (Figure 1B). On the test day, 5xFAD mice trans-passed the platform region at a lower frequency than WT (*p* = 0.4 for 4 months, *p* < 0.05 for 6 months, Figure 1C). This result indicates a deficit in spatial working memory in 5xFAD at 6 months.

After the water maze test, to determine whether the memory impairment of 5xFAD mice was also observed at the level of neuronal networks, we carried out hippocampal LTP recordings from the same batch of mice. Stimulating the Schaffer collaterals of CA3 elicited excitatory postsynaptic potentials (EPSPs) in the CA1 area (Figures 1D,E). After stable baseline recording in hippocampal slices of WT mice, a theta burst stimulation (TBS) induced long-term potentiation of EPSP in CA1 area. This LTP effect was hardly observed in 5xFAD mice at 6 months (Figure 1E).

### Aβ Plaque Deposits Are Detected in the Brain of 5xFAD at 4 Months

**Figure 2:**
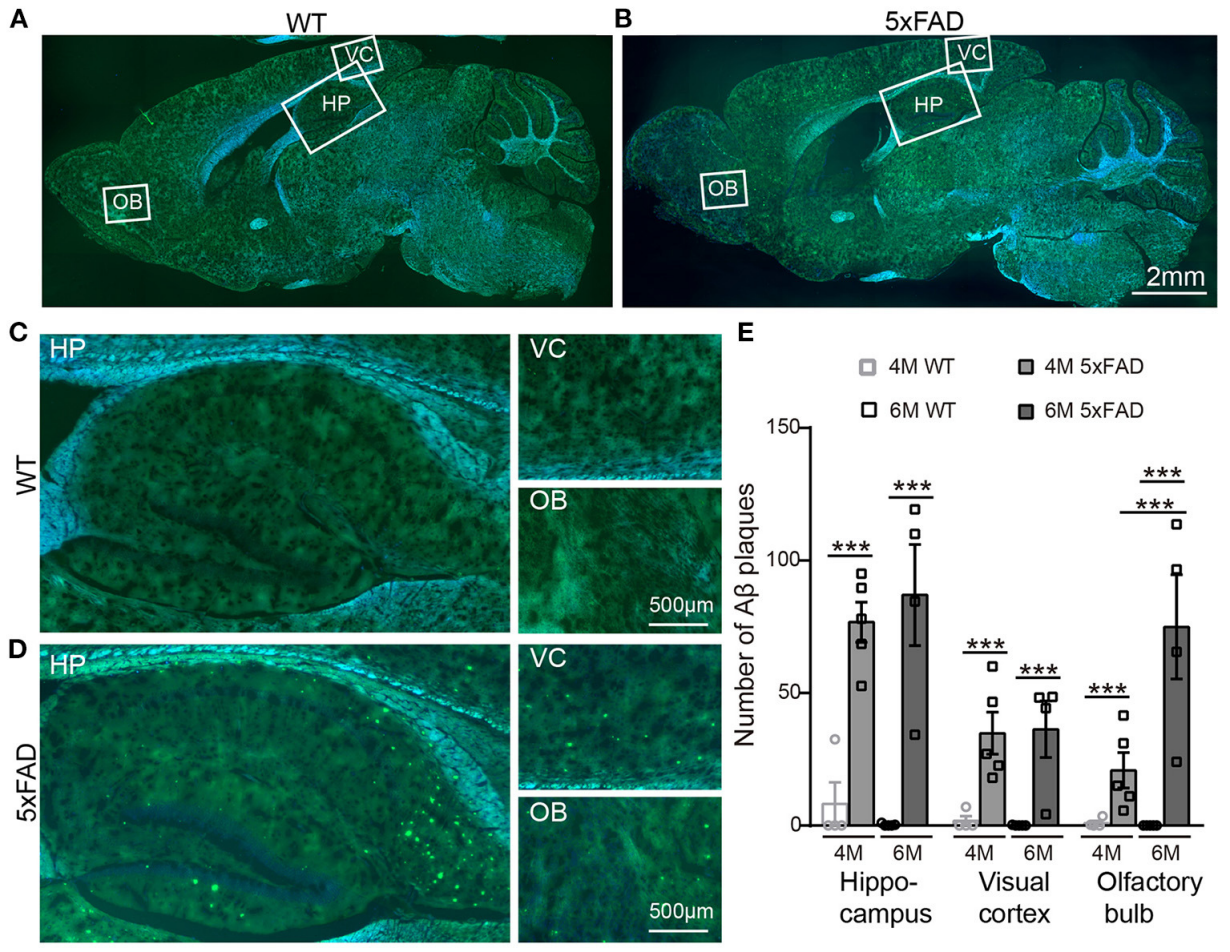
Aβ plaques appear in various brain regions of 5xFAD mice already at 4 months. **(A,B)** Images of brain slices stained with thioflavine S (green) that labels Aβ plaques from a **(A)** WT and **(B)** 5xFAD mice. Brain regions including olfactory bulb (OB), hippocampus (HP), and visual cortex (VC) were enlarged in Panels **(C)** and **(D)**, respectively, for WT and 5xFAD. **(E)** Number of Aβ plaques in different brain regions of WT and 5xFAD at 4 and 6 months. At 4 months, 5xFAD mice have more Aβ plaques in the brain than WT. Data shown as mean ± SEM. ***p < 0.001, two-way ANOVA test.

After identifying the memory and LTP deficits in 5xFAD, we tested the expression of Aβ plaques in different brain regions using thioflavine S staining. The staining showed that there were obvious Aβ plaques in the olfactory bulb (OB), the visual cortex (VC), and the hippocampus (HP) of 5xFAD mice at both 4 and 6 months (Figures 2A,B, with enlarged areas shown in Figures 2C,D), while no plaques were found in WT controls. The number of plaques in each brain region of 5xFAD was significantly higher than that in WT (Figure 2E). Interestingly, in the olfactory bulb of 5xFAD mice, the accumulation of Aβ plaque was more gradual as it increased from 4 to 6 months (21.9 ± 1.5 vs. 73.0 ± 5.0, *p* < 0.001), while the number in the hippocampal region and the visual cortex was already high at 4 months and then remained stable or increased slowly (Figure 2E).

### The Visual Behavior of 5xFAD Mice Is Impaired at 6 Months

After confirming the AD symptoms in the brain of 5xFAD mice, we wondered whether the visual system had similar deficits. We examined the visual system by optokinetic behavior and electroretinogram recordings and then by histological examinations.

**Figure 3:**
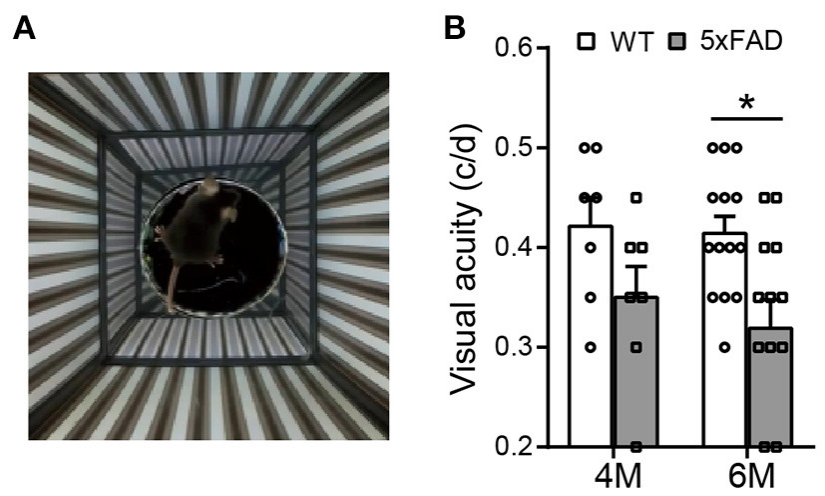
The visual acuity is impaired in 5xFAD mice at 6 months. **(A)** Illustration of the optokinetic system that measures the mouse visual acuity. Visual acuity is the maximum spatial frequency of the moving gratings that a mouse can track with its head movement. **(B)** Scattered plot of the visual acuity of 5xFAD and WT at 4 and 6 months. The visual acuity of 5xFAD was lower than that of WT both at 4 months and at 6 months, and the difference reached significance at 6 months. Data shown as mean ± SEM. *p < 0.05, two-way ANOVA.

The optokinetic system tests the ability of a mouse to track rotating gratings with its head (Figure 3A). The visual acuity (i.e., the highest spatial frequency of the grating that can induce the optokinetic reflex in mice) of the 5xFAD mouse decreased compared to WT, and the difference reached significance at 6 months (0.32 ± 0.03 c/d vs. 0.41 ± 0.02 c/d in WT, *p* < 0.05) but not at 4 months (Figure 3B).

The ability of a mouse to tell luminance was tested with a black/light transition box. The 5xFAD mice tended to spend shorter time than WT control in the black chamber, but the difference did not reach statistical significance at 6 months (5xFAD: 174 ± 23 s in black box, *n* = 4; WT: 208 ± 19 s, *n* = 6; not shown).

### Rod and Cone Light Responses Are Normal in 5xFAD Mice

**Figure 4:**
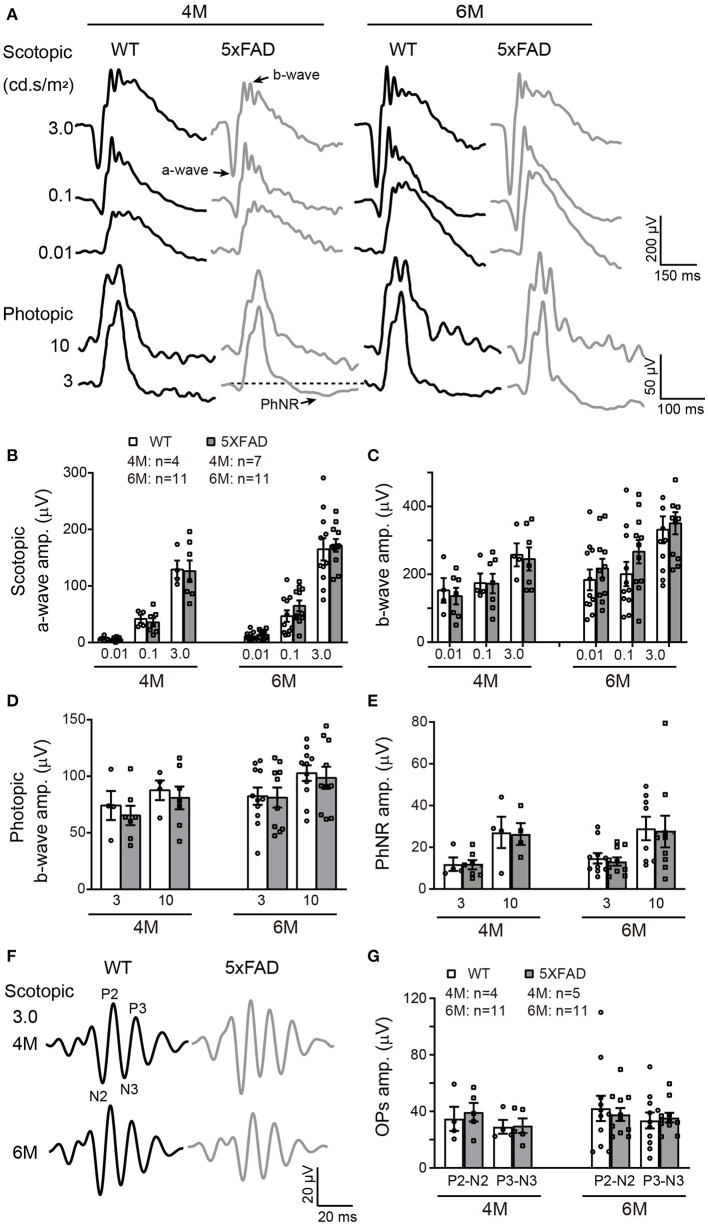
The light responses of the first and second order retinal neurons are normal in 5xFAD. **(A)** Example ERG traces from a WT and 5xFAD mice at age of 4 and 6 months under dark adaptation (scotopic) and light adaptation (photopic). **(B,C)** Scattered plot of the amplitude of **(B)** a-wave and **(C)** b-wave under scotopic condition. **(D,E)** Scattered plots of the amplitude of **(D)** b-wave and **(E)** PhNR under photopic condition. **(F)** Example OPs traces to flash of 3.0 cd s/m^2^ under scotopic condition. **(G)** Scattered plots of the amplitude of P2-N2 and P3-N3. Data shown as mean ± SEM.

The deficit in the above visual behavior indicates a deficit either in the retina or the retina-to-superior colliculus pathway (or both). Thus, we next evaluated the retinal function by full-field ERG recording. Under dark adaptation (scotopic conditions), both WT and 5xFAD mice responded well to flashes of increasing intensities (0.01, 0.1, and 3.0 cd s/m^2^) (Figure 4A). The amplitude of the a- and b-waves were similar between 5xFAD and WT at both 4 and 6 months (Figures 4B,C), indicating that the rod-to-rod bipolar pathway is normal in 5xFAD. We next light adapted the mice (photopic condition) and recorded cone responses. Again, the b-wave amplitude was similar between 5xFAD and WT at 4 and 6 months (Figures 4A,D), indicating that the light response of cones and bipolar cells are also normal in the 5xFAD mouse. The time-to-peak of a- and b-waves under both scotopic and photopic conditions were also similar between 5xFAD and WT (data not shown).

The light responses of amacrine cells were also evaluated by the oscillatory response (OPs) using ERG recordings. 5xFAD mice showed a normal OPs response as did WT at both 4 and 6 months (Figure 4F), and the amplitudes of P2-N2 and P3-N3 were similar to WT (Figure 4G).

### The Light Response of Retinal Ganglion Cell Is Reduced in 5xFAD

**Figure 5:**
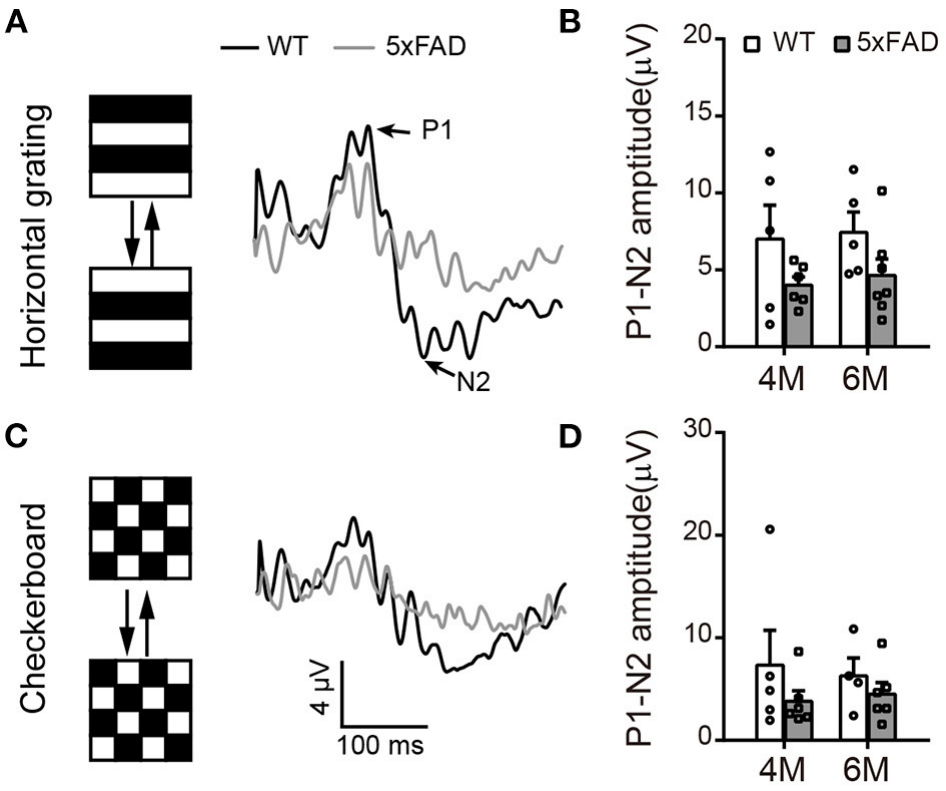
Pattern ERG response tend to decrease in 5xFAD. **(A,C)** Example traces of pattern ERG responses to **(A)** horizontal gratings or **(C)** checkerboard from a WT and 5xFAD mice. **(B,D)** Scattered plots of the P1-N2 amplitudes under stimuli of **(B)** horizontal gratings or **(D)** checkerboard. Data shown as mean ± SEM.

To check the light response of retinal ganglion cells, we first used ERG and measured the amplitude of the photopic negative response (PhNR, a negative wave that follows the b-wave under light adaptation) to full-field stimuli. The PhNR response was similar between 5xFAD and WT at both 4 and 6 months, with similar amplitude and time to peak (Figure 4E). Similar results were obtained with responses to pattern ERG, which is more sensitive (Liu et al., 2014). For that, we used patterned stimuli including horizontal grating and checkerboard (Figures 5A,C). Under both stimuli, 5xFAD mice tended to have smaller P1-N2 amplitude, but there was no significant difference (Figures 5B,D).

**Figure 6:**
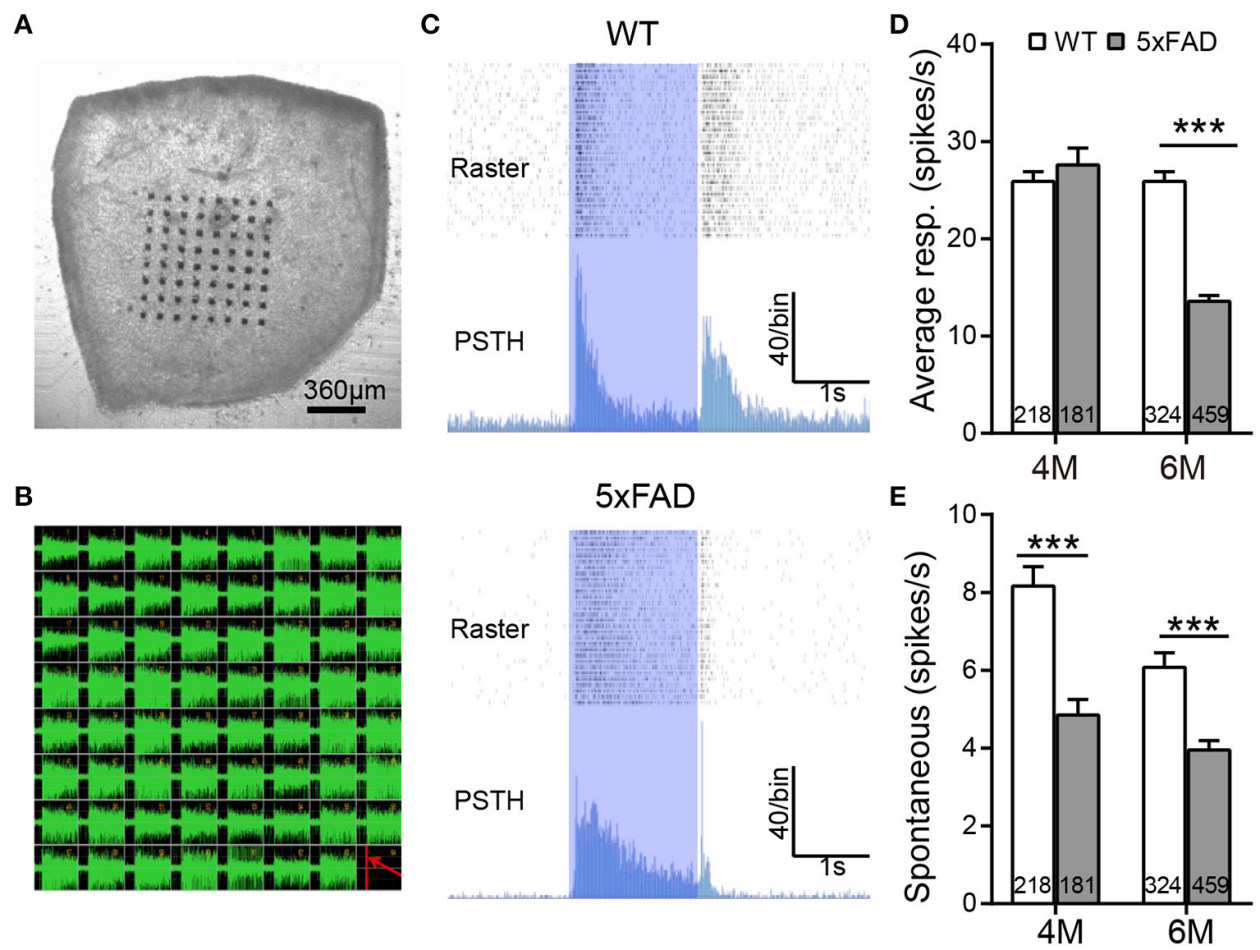
The light response of individual ganglion cells is reduced in 5xFAD mice at 6 months. **(A)** Illustration of an isolated retinal tissue placed on an 8×8 multielectrode array. **(B)** Spikes recorded from each electrode in response to a saturated flash (light intensity (4.68 × 10^7^ photons/μm^2^/s) whose onset is indicated by a red line in the lower right corner of the array. **(C)** Examples of spiking responses from WT and 5xFAD RGCs. For each cell, the top panel shows a raster plot from 30 repeats, and the bottom panel shows the corresponding PSTH. The 2-s light stimuli are indicated as blue regions. **(D)** Average firing rate of RGCs within the 2-s light stimuli was significantly reduced at 6 months in 5xFAD. **(E)** Spontaneous firing was greatly decreased in 5xFAD mice at both 4 and 6 months. The numbers within the bars represent the number of responsive cells recorded from retinas of three animals. ***p < 0.001, two-way ANOVA.

We further analyzed the light responses of individual ganglion cells by multielectrode array recordings. An example of a flattened retina mounted on 8×8 MEA array, and the spike responses recorded in each channel are shown in Figures 6A,B. Both WT and 5xFAD RGCs fired strongly in response to light stimuli (Figure 6C). Comparing the average light response within the 2-s flashes showed a significant decrease in 5xFAD at 6 months (to 52% of WT, *p* < 0.001) but not at 4 months (Figure 6D). Similar changes were observed in the peak firing rate (4 months: 100.8% of WT, *p* = 0.97; 6 months: 89% of WT, *p* < 0.001, data not shown). While the light responses were not much affected at 4 months, the spontaneous frequency of 5xFAD started to decrease significantly compared with WT already at 4 months (59% of WT at 4 months and 65% of WT at 6 months, *p* < 0.001) (Figure 6E).

### Retinal Ganglion Cells Appear Normal in 5xFAD Retina at 6 Months With a Few Aβ Plaques

**Figure 7:**
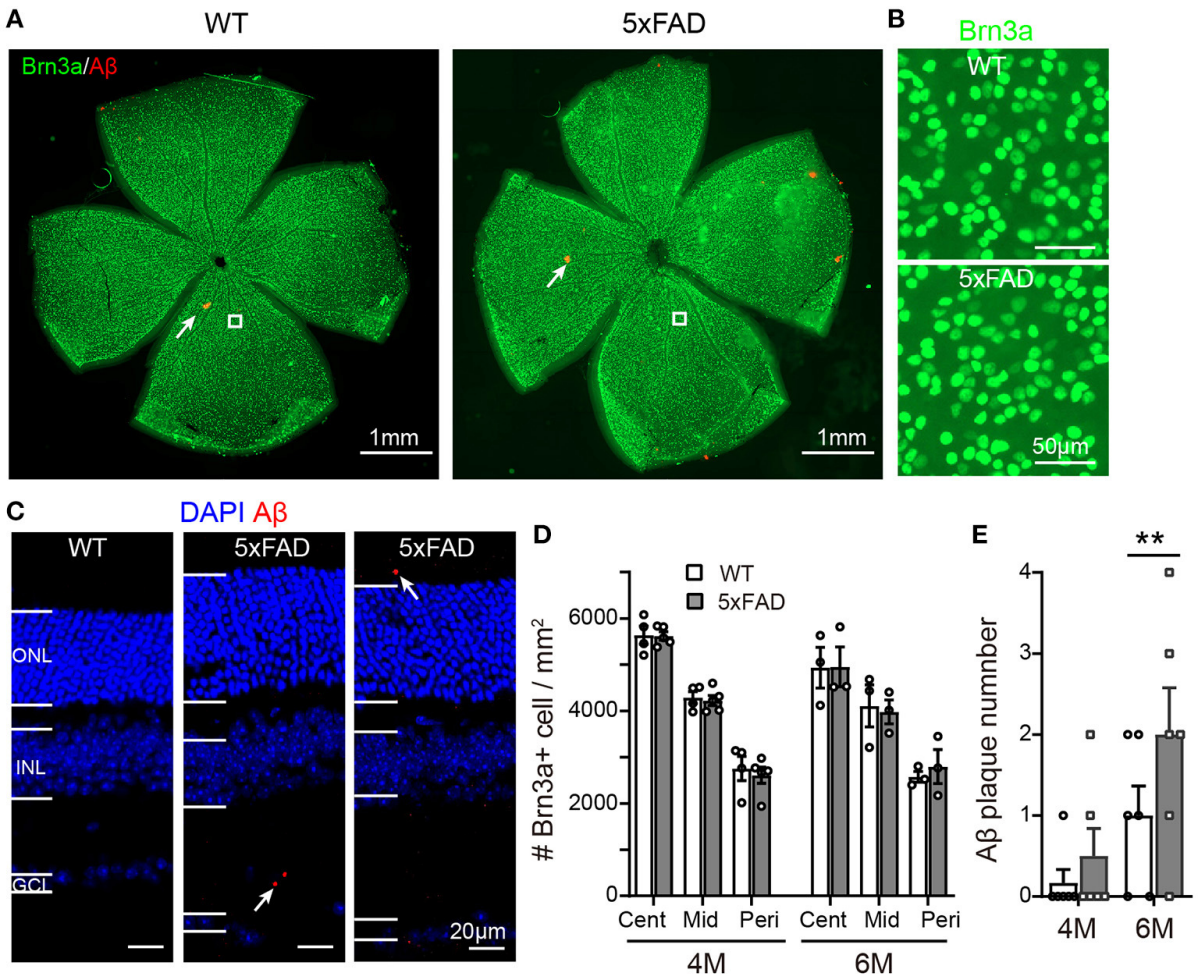
Density of retinal ganglion cells is normal in 5xFAD but a few Aβ plaques emerge in retina at 6 months. **(A)** Example of Brn3a staining of RGCs (green) and Aβ plaque deposition (red, examples indicated by white arrows) from a whole mount retina of a WT and 5xFAD mice at 6 months; the white square from similar eccentricity is shown in Panel **(B)** at higher magnification. **(C)** Images of retinal slices stained with DAPI and Aβ plaque deposition (red, points by white arrow). **(D)** Scattered plot of the Brn3a-positive cell density in whole mount retina at central (cent), middle (mid), and peripheral (peri) regions (corresponding to 300, 1,000, and 1,700 μm away from the center of the optic disk, respectively). The RGCs density in 5xFAD is similar to that of WT at both 4 and 6 months. **(E)** Scattered plot of Aβ plaque number in retinal slices (accumulated from three slices for each animal). **p < 0.01; two-way ANOVA.

As the function of ganglion cells in 5xFAD mice declined, we further examined the survival rate of RGCs by Brn3a staining on the whole-mount retina. The number of RGCs was counted for three eccentricities; for each eccentricity, we counted and averaged the numbers from four quadrants of the whole-mount retina (Figure 7A, enlarged area shown in Figure 7B). For each eccentricity, the number of RGCs in 5xFAD and WT at 4 months and 6 months was similar (Figures 7A,B,D).

We have also stained the retinas for Aβ plaque deposits with an Aβ1-42 antibody. For both WT and 5xFAD, staining in whole-mount retinas revealed only few Aβ plaques (red dots pointed by white arrows in Figure 7A). In retinal slices, however, a few Aβ deposits were observed also in the inner and outer retinal layers of 5xFAD mice but not that of WT (the sampled region was 1 mm from the optic disk) (Figure 7C). Counting the number of Aβ deposits in these retinal slices showed that 5xFAD retina has accumulated significantly more Aβ deposits than the WT controls (2.0 ± 0.2 accumulated from three retinal slices for each animal vs. 1.0 ± 0.2 in WT, *p* < 0.05) (Figure 7E).

### The Retina Becomes Abnormally Thick in 5xFAD Mice

**Figure 8:**
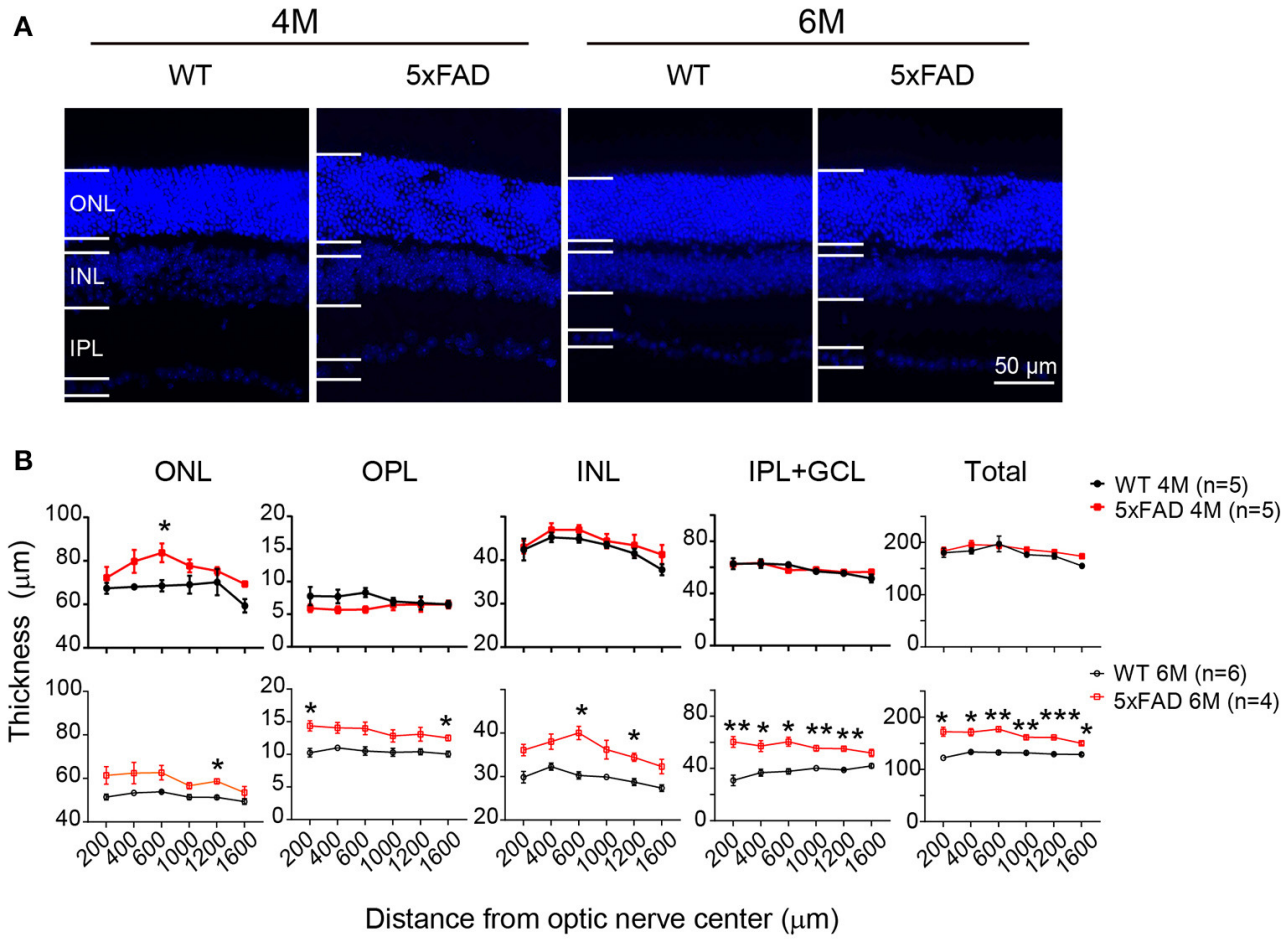
Retina is abnormally thick in 5xFAD mice already at 4 months. **(A)** Images of DAPI staining of retinal slices from a WT and 5xFAD mice at 4 and 6 months. **(B)** Retinal thickness from the center to periphery for ONL, OPL, INL, and IPL with GCL layers and the total thickness at 4 months (top panel) and 6 months (bottom panel). The retina of 5xFAD mice is thicker than that of WT at 6 months. ONL, outer nuclear layer; INL, inner nuclear layer; GCL, ganglion cell layer. Data shown as mean ± SEM. *p < 0.05, **p < 0.01; ***p < 0.001, two-way ANOVA with repetitive measurement. n, number of animals tested.

While examining the Aβ plaques, we noticed that the 5xFAD retina appeared thicker and less organized. We have therefore further examined the structure of the retinal layers. Using DAPI staining, we measured the thickness of each retinal layer from the center to periphery, with examples of images collected from the middle region (800–1,000 μm away from the optic disk center) shown in Figure 8A. At 4 months, all retinal layers except ONL in 5xFAD were as normal as WT (top panel, Figure 8B). For the ONL in 5xFAD, the thickness tended to increase from the center to middle region, then dropped in the peripheral region, and the difference from WT reached significance at the middle region of the retina. At the age of 6 months, all retinal layers of 5xFAD were thicker than WT, with regional limited thicker ONL, OPL, and INL, but a widespread thicker IPL, thus a general thicker retina from center-to-peripheral regions (bottom panel, Figure 8B).

### Retinal Glial Cells in Retina of 5xFAD at 6 Months Are Activated

Since pathological changes often accompany inflammation, we next examined retinal inflammation using the indicator of reactive gliosis in microglia and Muller cells.

**Figure 9:**
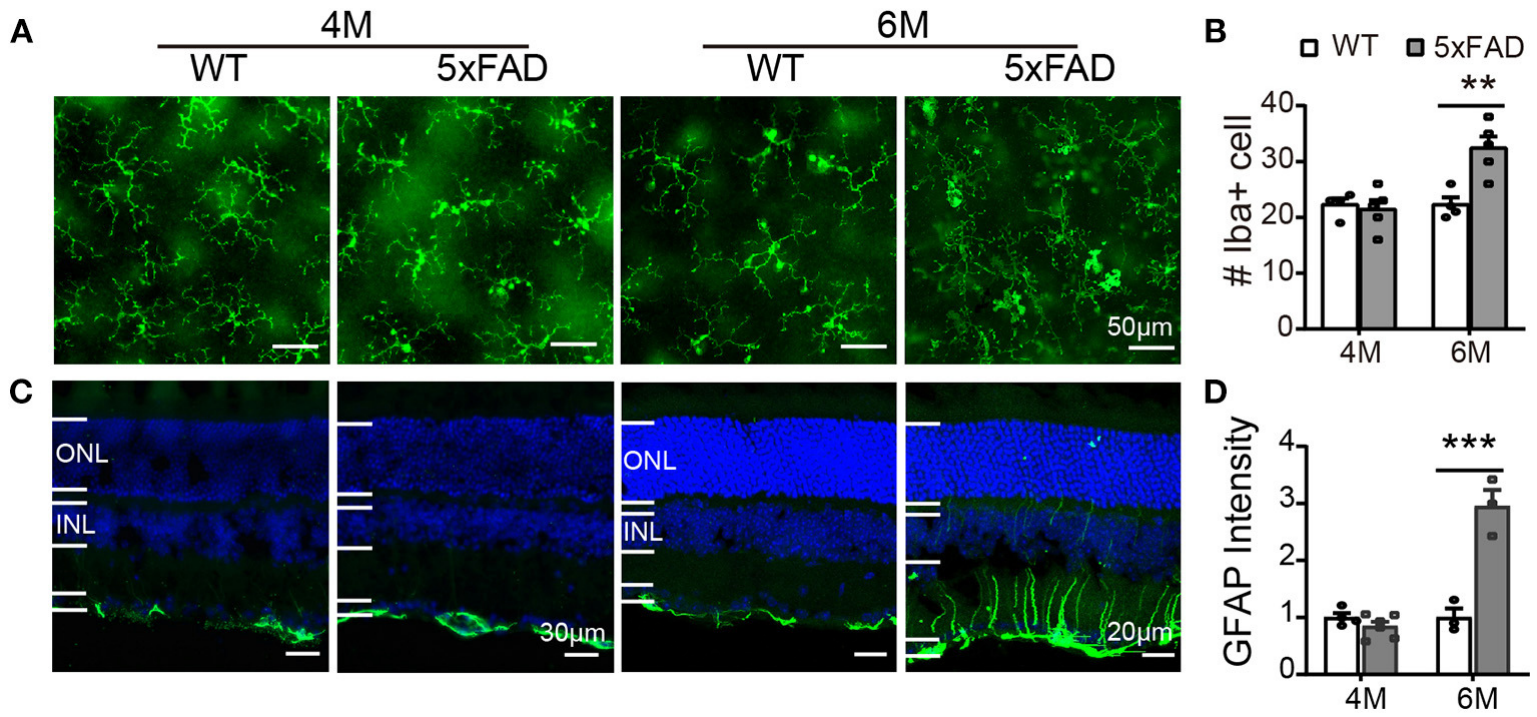
Reactive gliosis happens in 5xFAD at 6 months. **(A)** Images of Iba1 staining (green) on whole mount retinas of WT and 5xFAD mice at 4 and 6 months. **(B)** Number of Iba1+ cells on retina (an image size of 320×320 μm). **(C)** Images of GFAP staining (green) on retinal sections of WT and 5xFAD mice at both ages. Note the difference in scale. **(D)** The GFAP intensity in the inner retina, normalized to the WT control. ONL, outer nuclear layer; INL, inner nuclear layer; GCL, ganglion cell layer. Data shown as mean ± SEM. **p < 0.01; ***p < 0.001, two-way ANOVA.

In WT retina, microglia cells stained with Iba1 showed a resting state with small somas and many elongated protrusions extending around the soma. In 5xFAD retina, the appearance of the microglia cells was different at 6 months, with often shorter and less organized branches (Figure 9A), and the number significantly increased at 6 months (32.4 ± 3.7 vs. 22.3 ± 2.6 per 320 × 320 μm for WT, *p* < 0.01) (Figure 9B), indicating an active state. In WT retina, GFAP in Muller cells was limited to the end feet in the inner limiting layer, showing a resting state. In 5xFAD retina, the GFAP remained in the end feet area at 4 months, but at 6 months, the GFAP positive staining appeared in many processes that cross the retina (Figure 9C). The fluorescent intensity of GFAP in the inner retinal layers (INL, IPL, and GCL layers) increased significantly at 6 months (*p* < 0.01) (Figure 9D). We therefore conclude that glial cells in 5xFAD retinas are being activated at 6 months.

## Discussion

In this study, we examined the retinal structure and function of 5xFAD mice carrying no rd1 mutation and compared the temporal changes with the brain. Obvious retinal pathologies including thickening of retina, reactive gliosis, and few but significantly increased Aβ plaques were identified at 6 months old. An abnormal thickening of the outer nuclear layer was noticed already at 4 months. Functionally, full-field ERG and pERG remained normal, but visual acuity and light responses of individual RGCs were reduced at 6 months. In comparison, numerous Aβ plaques appeared in different regions of the brain at 4 months, but the working memory and hippocampal LTP significantly decayed only at 6 months. Thus, the timeline of retinal abnormality coincides with the progress of brain degeneration.

As early symptoms of AD include functional deficit of smell and vision, olfactory and vision biomarkers are suggested to serve as noninvasive biomarkers to diagnose dementia (Romano et al., 2021). In AD patients or AD mice, beta-amyloid deposition is found in the olfactory bulb [reviewed by Dibattista et al. (2020)]. In the 5xFAD mice, besides hippocampal region, we also found an accumulation of Aβ in the olfactory bulb and visual cortex, confirming the AD pathology in various brain regions in 5xFAD mice. But unlike visual cortex and hippocampus, the rise in Aβ deposition in the olfactory bulb was initially slow, and it kept on rising from 4 to 6 months. This suggests that the progression of AD pathology in olfactory bulb develops more slowly than other two brain regions. Indeed, a slower decay of the volume of olfactory bulb than hippocampus at the early and middle stages of AD was reported in rTg510 mice, another AD mice model (Kim et al., 2017). In another study on 5xFAD mice, an intact olfactory memory from 3 to 15 months of age was reported (O'Leary et al., 2020). Whether olfactory system decay differentially from hippocampus and visual system in AD mice may need further study, but it is not the main focus of current study.

Consistent with other reports, we found normal full-field ERG responses in 5xFAD both at 4 and 6 months, indicating unaffected photoreceptors and bipolar cells (Criscuolo et al., 2018; Lim et al., 2020). Regarding RGCs, we did not find an impairment of the amplitude of PhNR or pERG, which represents a compound light response of all RGCs (Porciatti, 2007; Chrysostomou and Crowston, 2013). However, those methods may not be as sensitive as the pSTR used by Lim et al., who reported a decreased amplitude of pSTR at 6 months on the same mouse line (Lim et al., 2020). This finding is consistent with our MEA data, which showed a reduced light response in RGCs in 5xFAD by 6 months and also a reduced spontaneous firing at 4 months. The dysfunction of RGCs was also reported in AD patient (Parisi et al., 2001). The response of amacrine cell, indicated by OPs, remained normal in 5xFAD as in WT. Thus, the reduction in ganglion cell activity found with MEA recordings probably depends on direct changes within these cells rather than abnormal transmission from their upstream cells.

Morphologically, instead of observing retinal thinning as in other reports on animals (Liu et al., 2009; Georgevsky et al., 2019) and AD patients [reviewed in Chiquita et al. (2019)], we found an abnormal thickening of the 5xFAD retina at 6 months, and the ONL was thicker already at 4 months. The seemingly different results are likely due to the different age used in these reports. In AD patients, the reduction in the retinal neve fiber layer (RNFL) is reported in patients showing mild to severe cognitive impairment (Paquet et al., 2007; Gao et al., 2015), while no difference or even thicker inner retina (especially IPL) in preclinical AD patients was also reported (Snyder et al., 2016; van de Kreeke et al., 2019). Consistent with the results from preclinical AD patient, Lim et al. observed a thickening of IPL at 6 months only, but not at a later stage (Lim et al., 2020) in 5xFAD mice, while RNFL layer decreased since 6 months. In other animal models such as Tg2576 mice (Liu et al., 2009), 3xTg mice (Song et al., 2020), and APP1/PS1 (Georgevsky et al., 2019), a thinning of the retinal layers was reported at late stages, but in an earlier study, using APP1/PS1 mice up to 12M, no retinal pathology was observed (Chidlow et al., 2017). The early thickening of the retina we observed in 5xFAD may be related to the inflammation that happened around 6 months or edema. Therefore, at the initial time, there might be edematous change before any detectable functional change. Future experiment using OCT system to check the retinal thickness in alive 5xFAD mice at early stages would help for early non-invasive diagnostic. Indeed, we were collecting a series of OCT scanning of 5xFAD retina together with its littermate at various time points (as early as 3M) to access this possibility. It may be puzzling that the ERG waves in 5xFAD mice were normal in spite of abnormal retinal thickness. This may be due to the full-field flash we applied, since full-field flash ERG averages responses all over the retina and may not be able to detect regional difference. Future experiment using multifocal ERG may help to identify any regional response abnormalities that may be correlated with the outer retinal structure.

In both AD patients and animal models, Aβ plagues were present in the retina (Koronyo-Hamaoui et al., 2011) [also see review Chiquita et al. (2019)]. Consistent with these reports, we found more Aβ plagues in 5xFAD retina than in WT, although the number of Aβ plagues was rather low even at 6 months, especially when compared with those in brain regions. In the other line of 5xFAD that carries rd1 mutation, Aβ present in the retina as early as 1.5M (Pogue et al., 2015). The earlier presence of Aβ in the retina may be due to the pathology caused by the rd1 mutation. Interestingly, besides 5xFAD, we also noticed a few Aβ staining on WT retina, and this was also mentioned by Barton et al. (2021) when they used inhalable thioflavin S (Barton et al., 2021). They also reported a significant association of Aβ deposits with RGCs. In 5xFAD mice (without rd1 mutation) age 6 months and older, extracellular Aβ plagues were found in ONL, INL, IPL, and GCL of the retina (Habiba et al., 2020). Consistent with this, in our study, we noticed that the few Aβ plagues appeared in the inner retinal layers as well as the outer retina. While Aβ plagues do accumulate in the retina, their accumulation may be too little to cause the abnormally thick retina we saw in 5xFAD. We believe that global inflammation (especially in the IPL where microglia got activated) may be the main reason at this age. In older animals, when numerous Aβ aggregates starts to appear in the retina, degradation of cells and synaptic proteins may happen and cause retinal degeneration. Indeed, Habiba et al. (2020) reported an increased number of Aβ plagues in aging 5xFAD mice at 12 and 17M, and the retina degenerated with age (Habiba et al., 2020). Currently, it is not known whether the synaptic proteins degrade in the aging 5xFAD mice as Aβ plague accumulates; further experiments on older AD mice may be needed to check this.

Using the same mouse line of 5xFAD carrying no rd1 mutation, Lim et al. (2020) found deficits in retinal function and structure at 6 months, the earliest time point they assessed. We also noticed retinal deficits at 6 months and further extended their study by exploring an earlier time point at 4 months. The retinal structure and function were in general normal at 4 months except a thickening of the outer nuclear layer in the middle region. The pathology in retina does not happen earlier than the pathology in brain, bringing the concern of using retinal pathology as an early preclinical marker of cortical and behavioral changes. However, obvious retinal deficits were noticed at 6 months when working memory and LTP significantly decay, so retinal abnormality in this line of 5xFAD concurred with brain degeneration. Thus, we suggest that using retinal pathology to reflect the changes in the brain is still the right way for AD diagnostic and evaluation of the treatment effects.

## Data Availability Statement

The raw data supporting the conclusions of this article will be made available by the authors, without undue reservation.

## Ethics Statement

The animal study was reviewed and approved by the ethics committee at Jinan University.

## Author Contributions

YX and KC designed the study. MZ carried out the major experiments including LTP recording, MEA recording, immunostaining, pattern ERG recording, behavioral tests, and data analysis. LZ and GX carried out the full-field ERG recording and analyzed the ERG data. XH, HC, SZ, and DX assisted the experiment and data analysis. MZ and YX wrote the manuscript and KC edited it. All authors contributed to manuscript revision, read, and approved the submitted version.

## Conflict of Interest

The authors declare that the research was conducted in the absence of any commercial or financial relationships that could be construed as a potential conflict of interest.

## Publisher's Note

All claims expressed in this article are solely those of the authors and do not necessarily represent those of their affiliated organizations, or those of the publisher, the editors and the reviewers. Any product that may be evaluated in this article, or claim that may be made by its manufacturer, is not guaranteed or endorsed by the publisher.
